# 4-[(9-Ethyl-9*H*-carbazol-3-yl)imino­meth­yl]phenol

**DOI:** 10.1107/S1600536810046167

**Published:** 2010-11-17

**Authors:** Songzhu Lin, Ruokun Jia, Xiaoli Gao, Haihui Yu, Yanlin Yuan

**Affiliations:** a Northeast China Electric Power University Personnel, Jilin 132012, People’s Republic of China

## Abstract

In the title compound, C_21_H_18_N_2_O, the dihedral angle between the phenol ring and the carbazole system is 39.34 (2)°. Inter­molecular O—H⋯N hydrogen bonds and C—H⋯π and π–π inter­actions [centroid–centroid distances = 3.426 (2) and 3.768 (2) Å] stabilize the crystal structure.

## Related literature

For polar organic mol­ecules as components of non-linear optical, electro-optical, photorefractive and optical-limiting materials, see: Nalwa & Miyata (1997 ▶); Kuzyk & Dirk (1998 ▶); Nesterov *et al.* (2002 ▶). 
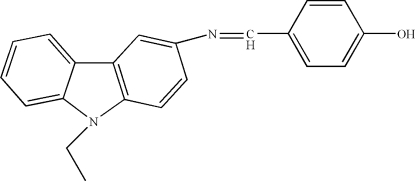


## Experimental

### 

#### Crystal data

C_21_H_18_N_2_O
*M* *_r_* = 314.37
Orthorhombic, 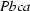

*a* = 13.386 (6) Å
*b* = 9.247 (4) Å
*c* = 26.443 (10) Å
*V* = 3273 (2) Å^3^
*Z* = 8
Mo *K*α radiation
μ = 0.08 mm^−1^
*T* = 295 K
0.25 × 0.20 × 0.15 mm

#### Data collection

Enraf–Nonius CAD-4 diffractometer
21605 measured reflections
2878 independent reflections
1615 reflections with *I* > 2σ(*I*)
*R*_int_ = 0.081
3 standard reflections every 100 reflections  intensity decay: none

#### Refinement

*R*[*F* ^2^ > 2σ(*F* ^2^)] = 0.050
*wR*(*F* ^2^) = 0.140
*S* = 1.02
2878 reflections
218 parameters
H-atom parameters constrained
Δρ_max_ = 0.18 e Å^−3^
Δρ_min_ = −0.18 e Å^−3^

### 

Data collection: *CAD-4 Software* (Enraf–Nonius, 1989 ▶); cell refinement: *CAD-4 Software*; data reduction: *NRCVAX* (Gabe *et al.*, 1989 ▶); program(s) used to solve structure: *SHELXS97* (Sheldrick, 2008 ▶); program(s) used to refine structure: *SHELXL97* (Sheldrick, 2008 ▶); molecular graphics: *SHELXTL* (Sheldrick, 2008 ▶); software used to prepare material for publication: *WinGX* (Farrugia, 1999 ▶).

## Supplementary Material

Crystal structure: contains datablocks global, I. DOI: 10.1107/S1600536810046167/vm2053sup1.cif

Structure factors: contains datablocks I. DOI: 10.1107/S1600536810046167/vm2053Isup2.hkl

Additional supplementary materials:  crystallographic information; 3D view; checkCIF report

## Figures and Tables

**Table 1:** Hydrogen-bond geometry (Å, °) *Cg*2 is the centroid of the C3–C8 ring.

*D*—H⋯*A*	*D*—H	H⋯*A*	*D*⋯*A*	*D*—H⋯*A*
O1—H1⋯N2^i^	0.82	2.09	2.842 (3)	153
C1—H1*A*⋯*Cg*2^ii^	0.96	2.77	3.698 (2)	162

Symmetry codes: (i) 
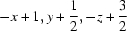
; (ii) 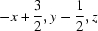.

**Fig. 1.**
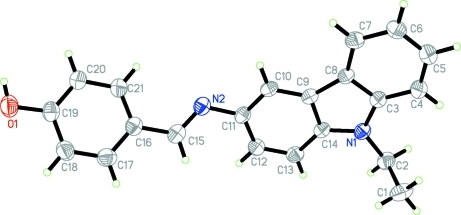
The structure of the title compound showing 30% probability displacement ellipsoids and the atom-numbering scheme.

## supplementary crystallographic information

### Comment

Polar organic molecules as components of NLO, electro-optical, photorefractive and optical-limiting materials have been under intensive investigation (Nalwa & Miyata, 1997; Kuzyk & Dirk, 1998; Nesterov *et al.*, 2002). Many *N*-ethylcarbazole derivatives have been studied for this purpose. In this paper, we describe the synthesis and structure determination of the title compound.

In the title compound, atoms O1, C15, N2 lie in the plane of phenyl ring C16—C21 (p1) with the largest deviation of 0.002 (3) Å for C16. The atoms of the carbazole ring together with C2 and N2 form a plane (p2) for which the largest deviation is 0.068 (1) Å for C5. The fragment C11,N2, C15,C16,C17 is coplanar (p3). The dihedral angles formed by p1 with p2 and p3 are 39.34 (2) and 6.01 (2)°, respectively. The dihedral angle between p2 and p3 is 42.21 (3)°.

In the lattice, π–π and C—H···π interactions occur [*Cg*1···*Cg*1^i^ = 3.426 (2), *Cg*2···*Cg*3^i^ = 3.768 (2) Å, C1···*Cg*2^ii^ = 3.698 (2) Å, H1A···*Cg*2^ii^ = 2.77 Å, symmetry codes: ^i^ 1 - *x*, -*y*, 1 - *z*; ^ii^ 3/2 - *x*, -1/2 + *y*, *z*. *Cg*1, *Cg*2, *Cg*3 refer to ring N1—C3—C8—C9—C14 and phenyl rings C3—C8 and C9—C14, respectively]. In addition, an intermolecular hydrogen bond (Table 1) along with the C—H···π and π–π interactions stabilizes the crystal structure. The H-bond results in infinite chains along [010].

### Experimental

The title compound was synthesized by reaction of 9-ethyl-carbazol-3-amine (0.420 g, 0.002 mol) and 4-hydroxybenzaldehyde (0.244 g, 0.002 mol) in ethanol (50 ml) under stirring for 5 h at room temperature. Single crystals suitable for *x*-ray measurements were obtained by recrystallization from ethyl acetate at room temperature.

### Refinement

H atoms were fixed geometrically and allowed to ride on their attached atoms, with C—H distances=0.93–0.96 Å, O—H distance=0.82 Å and with *U*_iso_=1.2–1.5*U*_eq_.

### Crystal data

**Table:**

C_21_H_18_N_2_O	*F*(000) = 1328
*M**_r_* = 314.37	*D*_x_ = 1.276 Mg m^−^^3^
Orthorhombic, *P**b**c**a*	Mo *K*α radiation, λ = 0.71073 Å
Hall symbol: -P 2ac 2ab	Cell parameters from 25 reflections
*a* = 13.386 (6) Å	θ = 4–14°
*b* = 9.247 (4) Å	µ = 0.08 mm^−^^1^
*c* = 26.443 (10) Å	*T* = 295 K
*V* = 3273 (2) Å^3^	Block, brown
*Z* = 8	0.25 × 0.20 × 0.15 mm

### Data collection

**Table:**

Enraf–Nonius CAD-4 diffractometer	*R*_int_ = 0.081
Radiation source: fine-focus sealed tube	θ_max_ = 25.0°, θ_min_ = 1.5°
graphite	*h* = −15→15
ω scans	*k* = −10→10
21605 measured reflections	*l* = −31→28
2878 independent reflections	3 standard reflections every 100 reflections
1615 reflections with *I* > 2σ(*I*)	intensity decay: none

### Refinement

**Table:**

Refinement on *F*^2^	Secondary atom site location: difference Fourier map
Least-squares matrix: full	Hydrogen site location: inferred from neighbouring sites
*R*[*F*^2^ > 2σ(*F*^2^)] = 0.050	H-atom parameters constrained
*wR*(*F*^2^) = 0.140	*w* = 1/[σ^2^(*F*_o_^2^) + (0.0568*P*)^2^ + 0.6129*P*] where *P* = (*F*_o_^2^ + 2*F*_c_^2^)/3
*S* = 1.02	(Δ/σ)_max_ < 0.001
2878 reflections	Δρ_max_ = 0.18 e Å^−^^3^
218 parameters	Δρ_min_ = −0.18 e Å^−^^3^
0 restraints	Extinction correction: *SHELXL97* (Sheldrick, 2008), Fc^*^=kFc[1+0.001xFc^2^λ^3^/sin(2θ)]^-1/4^
Primary atom site location: structure-invariant direct methods	Extinction coefficient: 0.0037 (8)

### Special details

**Table:**

Geometry. All e.s.d.'s (except the e.s.d. in the dihedral angle between two l.s. planes) are estimated using the full covariance matrix. The cell e.s.d.'s are taken into account individually in the estimation of e.s.d.'s in distances, angles and torsion angles; correlations between e.s.d.'s in cell parameters are only used when they are defined by crystal symmetry. An approximate (isotropic) treatment of cell e.s.d.'s is used for estimating e.s.d.'s involving l.s. planes.
Refinement. Refinement of *F*^2^ against ALL reflections. The weighted *R*-factor *wR* and goodness of fit *S* are based on *F*^2^, conventional *R*-factors *R* are based on *F*, with *F* set to zero for negative *F*^2^. The threshold expression of *F*^2^ > σ(*F*^2^) is used only for calculating *R*-factors(gt) *etc*. and is not relevant to the choice of reflections for refinement. *R*-factors based on *F*^2^ are statistically about twice as large as those based on *F*, and *R*- factors based on ALL data will be even larger.

### Fractional atomic coordinates and isotropic or equivalent isotropic displacement parameters (Å^2^)

**Table:**

	*x*	*y*	*z*	*U*_iso_*/*U*_eq_	
O1	0.61757 (15)	0.7932 (2)	0.83217 (7)	0.0802 (7)	
H1	0.5624	0.8316	0.8314	0.096*	
N1	0.64140 (15)	0.0252 (2)	0.47293 (8)	0.0538 (6)	
N2	0.58786 (17)	0.3455 (2)	0.64785 (8)	0.0580 (6)	
C1	0.7978 (2)	−0.0332 (4)	0.42951 (13)	0.0866 (10)	
H1A	0.8397	−0.1123	0.4193	0.130*	
H1B	0.8343	0.0292	0.4519	0.130*	
H1C	0.7772	0.0202	0.4002	0.130*	
C2	0.7074 (2)	−0.0908 (3)	0.45633 (11)	0.0639 (8)	
H2B	0.6711	−0.1547	0.4338	0.077*	
H2C	0.7285	−0.1468	0.4855	0.077*	
C3	0.57518 (18)	0.0966 (3)	0.44114 (10)	0.0509 (7)	
C4	0.5568 (2)	0.0753 (3)	0.39037 (11)	0.0635 (8)	
H4A	0.5892	0.0024	0.3725	0.076*	
C5	0.4897 (2)	0.1644 (3)	0.36707 (12)	0.0710 (9)	
H5A	0.4767	0.1523	0.3328	0.085*	
C6	0.4407 (2)	0.2721 (3)	0.39335 (12)	0.0692 (8)	
H6A	0.3953	0.3310	0.3765	0.083*	
C7	0.45795 (19)	0.2937 (3)	0.44390 (11)	0.0605 (7)	
H7A	0.4241	0.3658	0.4614	0.073*	
C8	0.52679 (18)	0.2060 (3)	0.46856 (10)	0.0473 (6)	
C9	0.56752 (17)	0.2025 (3)	0.51875 (9)	0.0462 (6)	
C10	0.55122 (17)	0.2834 (3)	0.56227 (10)	0.0509 (7)	
H10A	0.5030	0.3558	0.5624	0.061*	
C11	0.60673 (19)	0.2563 (3)	0.60528 (10)	0.0518 (7)	
C12	0.6761 (2)	0.1429 (3)	0.60559 (10)	0.0576 (7)	
H12A	0.7122	0.1242	0.6349	0.069*	
C13	0.69205 (19)	0.0585 (3)	0.56350 (11)	0.0579 (7)	
H13A	0.7376	−0.0174	0.5642	0.069*	
C14	0.63826 (18)	0.0899 (3)	0.51982 (10)	0.0492 (7)	
C15	0.6605 (2)	0.3883 (3)	0.67601 (11)	0.0613 (8)	
H15A	0.7244	0.3539	0.6692	0.074*	
C16	0.6471 (2)	0.4883 (3)	0.71814 (10)	0.0593 (7)	
C17	0.7279 (2)	0.5339 (3)	0.74665 (11)	0.0716 (9)	
H17A	0.7907	0.4947	0.7404	0.086*	
C18	0.7171 (2)	0.6370 (4)	0.78447 (11)	0.0741 (9)	
H18A	0.7724	0.6675	0.8029	0.089*	
C19	0.6242 (2)	0.6938 (3)	0.79452 (10)	0.0624 (8)	
C20	0.5425 (2)	0.6490 (3)	0.76697 (11)	0.0685 (8)	
H20A	0.4795	0.6866	0.7739	0.082*	
C21	0.5548 (2)	0.5481 (3)	0.72916 (11)	0.0684 (8)	
H21A	0.4994	0.5192	0.7105	0.082*	

### Atomic displacement parameters (Å^2^)

**Table:**

	*U*^11^	*U*^22^	*U*^33^	*U*^12^	*U*^13^	*U*^23^
O1	0.0871 (15)	0.0854 (15)	0.0680 (13)	−0.0061 (12)	0.0009 (11)	−0.0171 (12)
N1	0.0561 (13)	0.0473 (13)	0.0581 (15)	0.0069 (11)	0.0047 (11)	−0.0006 (11)
N2	0.0668 (15)	0.0555 (14)	0.0516 (14)	0.0026 (12)	0.0064 (12)	0.0043 (12)
C1	0.0619 (19)	0.094 (2)	0.104 (3)	0.0178 (18)	0.0165 (18)	0.007 (2)
C2	0.0691 (18)	0.0502 (17)	0.072 (2)	0.0120 (15)	0.0020 (15)	−0.0050 (15)
C3	0.0504 (15)	0.0465 (15)	0.0558 (18)	−0.0040 (13)	0.0049 (14)	0.0011 (14)
C4	0.0670 (18)	0.0630 (19)	0.061 (2)	0.0038 (16)	0.0000 (15)	−0.0093 (16)
C5	0.074 (2)	0.080 (2)	0.0587 (19)	0.0030 (18)	−0.0070 (16)	−0.0039 (17)
C6	0.0697 (19)	0.073 (2)	0.065 (2)	0.0118 (16)	−0.0099 (16)	0.0089 (17)
C7	0.0571 (17)	0.0580 (18)	0.066 (2)	0.0058 (14)	0.0018 (15)	0.0063 (16)
C8	0.0452 (14)	0.0447 (15)	0.0521 (17)	−0.0003 (12)	0.0060 (12)	0.0034 (13)
C9	0.0459 (14)	0.0403 (14)	0.0524 (17)	0.0001 (12)	0.0066 (12)	0.0055 (13)
C10	0.0479 (15)	0.0446 (15)	0.0601 (17)	0.0040 (12)	0.0078 (14)	0.0049 (14)
C11	0.0564 (16)	0.0490 (16)	0.0499 (17)	0.0001 (13)	0.0109 (14)	0.0028 (14)
C12	0.0619 (17)	0.0568 (17)	0.0540 (18)	0.0030 (15)	−0.0014 (14)	0.0062 (15)
C13	0.0585 (17)	0.0518 (16)	0.0633 (19)	0.0102 (13)	−0.0003 (15)	0.0057 (15)
C14	0.0484 (15)	0.0434 (15)	0.0557 (18)	−0.0011 (13)	0.0075 (13)	0.0001 (14)
C15	0.0726 (19)	0.0539 (17)	0.0573 (18)	0.0054 (16)	0.0096 (16)	0.0085 (15)
C16	0.0678 (19)	0.0598 (18)	0.0502 (18)	0.0014 (15)	0.0049 (15)	0.0060 (14)
C17	0.063 (2)	0.081 (2)	0.071 (2)	0.0010 (17)	0.0020 (16)	0.0031 (19)
C18	0.072 (2)	0.087 (2)	0.063 (2)	−0.0078 (18)	−0.0039 (16)	−0.0078 (18)
C19	0.075 (2)	0.0636 (18)	0.0484 (17)	−0.0049 (17)	0.0060 (16)	0.0014 (15)
C20	0.069 (2)	0.075 (2)	0.0622 (19)	0.0049 (16)	0.0005 (16)	−0.0105 (17)
C21	0.068 (2)	0.078 (2)	0.0593 (19)	0.0006 (17)	0.0005 (15)	−0.0099 (17)

### Geometric parameters (Å, °)

**Table:**

O1—C19	1.358 (3)	C8—C9	1.435 (3)
O1—H1	0.8200	C9—C10	1.390 (3)
N1—C14	1.377 (3)	C9—C14	1.407 (3)
N1—C3	1.389 (3)	C10—C11	1.382 (3)
N1—C2	1.457 (3)	C10—H10A	0.9300
N2—C15	1.288 (3)	C11—C12	1.401 (3)
N2—C11	1.418 (3)	C12—C13	1.376 (3)
C1—C2	1.500 (4)	C12—H12A	0.9300
C1—H1A	0.9600	C13—C14	1.392 (3)
C1—H1B	0.9600	C13—H13A	0.9300
C1—H1C	0.9600	C15—C16	1.459 (4)
C2—H2B	0.9700	C15—H15A	0.9300
C2—H2C	0.9700	C16—C17	1.384 (4)
C3—C4	1.379 (3)	C16—C21	1.385 (4)
C3—C8	1.403 (3)	C17—C18	1.389 (4)
C4—C5	1.366 (4)	C17—H17A	0.9300
C4—H4A	0.9300	C18—C19	1.376 (4)
C5—C6	1.380 (4)	C18—H18A	0.9300
C5—H5A	0.9300	C19—C20	1.378 (4)
C6—C7	1.371 (4)	C20—C21	1.377 (4)
C6—H6A	0.9300	C20—H20A	0.9300
C7—C8	1.390 (3)	C21—H21A	0.9300
C7—H7A	0.9300		
			
C19—O1—H1	109.5	C14—C9—C8	106.9 (2)
C14—N1—C3	108.6 (2)	C11—C10—C9	120.0 (2)
C14—N1—C2	127.6 (2)	C11—C10—H10A	120.0
C3—N1—C2	123.7 (2)	C9—C10—H10A	120.0
C15—N2—C11	120.2 (2)	C10—C11—C12	119.8 (2)
C2—C1—H1A	109.5	C10—C11—N2	116.9 (2)
C2—C1—H1B	109.5	C12—C11—N2	123.3 (2)
H1A—C1—H1B	109.5	C13—C12—C11	121.5 (2)
C2—C1—H1C	109.5	C13—C12—H12A	119.2
H1A—C1—H1C	109.5	C11—C12—H12A	119.2
H1B—C1—H1C	109.5	C12—C13—C14	118.2 (2)
N1—C2—C1	111.7 (2)	C12—C13—H13A	120.9
N1—C2—H2B	109.3	C14—C13—H13A	120.9
C1—C2—H2B	109.3	N1—C14—C13	129.8 (2)
N1—C2—H2C	109.3	N1—C14—C9	108.9 (2)
C1—C2—H2C	109.3	C13—C14—C9	121.3 (2)
H2B—C2—H2C	107.9	N2—C15—C16	122.9 (3)
C4—C3—N1	129.4 (2)	N2—C15—H15A	118.5
C4—C3—C8	121.6 (3)	C16—C15—H15A	118.5
N1—C3—C8	109.0 (2)	C17—C16—C21	117.5 (3)
C5—C4—C3	118.0 (3)	C17—C16—C15	120.8 (3)
C5—C4—H4A	121.0	C21—C16—C15	121.6 (3)
C3—C4—H4A	121.0	C16—C17—C18	121.3 (3)
C4—C5—C6	121.4 (3)	C16—C17—H17A	119.4
C4—C5—H5A	119.3	C18—C17—H17A	119.4
C6—C5—H5A	119.3	C19—C18—C17	119.7 (3)
C7—C6—C5	121.1 (3)	C19—C18—H18A	120.2
C7—C6—H6A	119.5	C17—C18—H18A	120.2
C5—C6—H6A	119.5	O1—C19—C18	117.3 (3)
C6—C7—C8	118.9 (3)	O1—C19—C20	122.6 (3)
C6—C7—H7A	120.5	C18—C19—C20	120.0 (3)
C8—C7—H7A	120.5	C21—C20—C19	119.5 (3)
C7—C8—C3	119.0 (3)	C21—C20—H20A	120.2
C7—C8—C9	134.3 (2)	C19—C20—H20A	120.2
C3—C8—C9	106.6 (2)	C20—C21—C16	122.0 (3)
C10—C9—C14	119.2 (2)	C20—C21—H21A	119.0
C10—C9—C8	133.9 (2)	C16—C21—H21A	119.0

### Hydrogen-bond geometry (Å, °)

**Table:**

Cg2 is the centroid of the C3–C8 ring.

**Table:**

*D*—H···*A*	*D*—H	H···*A*	*D*···*A*	*D*—H···*A*
O1—H1···N2^i^	0.82	2.09	2.842 (3)	153
C1—H1A···Cg2^ii^	0.96	2.77	3.698 (2)	162

Symmetry codes: (i) −*x*+1, *y*+1/2, −*z*+3/2; (ii) −*x*+3/2, *y*−1/2, *z*.
